# Magnitude and associated factors of VIA positive test results for cervical cancer screening among refugee women aged 25–49 years in North Ethiopia

**DOI:** 10.1186/s12885-020-07344-9

**Published:** 2020-09-07

**Authors:** Gebretsadik Hailemariam, Hailay Gebreyesus, Tewolde Wubayehu, Tsgehana Gebregyorgis, Kidanemariam Gebrecherkos, Mebrahtu Teweldemedhin, Manaye Kifle

**Affiliations:** 1 Adminstration for Refugee and Returnee Affairs Adiharsh Eritrean refugee camps Health and Neutrino Coordinator, Adiharsh camp, Shire, Ethiopia; 2 Department of Public Health, College of Health Science, Aksum University, Aksum, Ethiopia; 3 School of Medicine, College of Health Science, Aksum University, Aksum, Ethiopia; 4 Department of Medical Laboratory Sciences, College of Health Science, Aksum University, Aksum, Ethiopia

**Keywords:** VIA positive, Precancerous cervical lesions, Refugee women

## Abstract

**Background:**

Worldwide cervical cancer is the third most common malignancy in women. It usually arises from the cervical area which is susceptible to Human Papilloma virus induced malignancy changes. In low-resource setting visual inspection with acetic acid (VIA) is an alternative sensitive cervical screening method. Therefore the aim of this study was to assess the magnitude and associated factors of VIA positive test results for Cervical Cancer screening among Eritrean refugee women aged 25–49 years in northern Ethiopia refugee camps.

**Methods:**

A community based cross-sectional study was conducted among 412 Eritrean refugee women aged 25–49 years from august 10 to September 25, 2018. Study subjects were selected by simple random sampling method. Data were collected using pretested structured questioner through Face-to-face interview and cervical examination. Data were coded and entered to Epi info software version 7 and then exported to Statistical package for Social Science (SPSS) version 21 for analysis. Bivariable and multivariable logistic regression analysis was made to test the association between the independent variables and the outcome variable. *P*-value of less than 0.05 with 95% CI was considered to declare statistical significance.

**Result:**

In this study the magnitude of VIA positive precancerous cervical lesions was 9% (95% CI: 6.3–11.8%). Previous history of sexually transmitted infections (STI) [AOR (95%CI) = 2.84(1.07–7.53)] and presence of STI during cervical examination [AOR (95%CI) =3.97(1.75–9.00)] were found significantly associated with VIA positive precancerous cervical lesions.

**Conclusions:**

In this study the magnitude of VIA positive precancerous cervical lesions was high. Previous history of sexually transmitted infections (STI) and presence of STI during cervical examination were found associated with VIA positive precancerous cervical lesions. Efforts such as early screening for sexually transmitted disease shall be done to prevent precancerous cervical lesions.

## Background

Cervical cancer (CC) is uncontrolled multiplication of normal cells of the cervix that arises from the squamous columnar junction (SCJ) [1]. It is mostly caused by persistent human papilloma virus (HPV) infection which causes precancerous cervical intraepithelial neoplasia (CIN) and CC [2]. The two most cancer causing HPV types are HPV16 & 18, which are responsible for 70% of CC and about 50% of CIN3 [3] and usually detected at around 25–30 years of age or about 10 years after first sexual intercourse [2]. Cervical cancer remained the leading cause of cancer related death for women in developing countries [4]. At the early stage, the most common finding in patients with CC is abnormal Pap test result or positive VIA [5]. After confirmation, CC cases can be managed with surgery or radiotherapy [6].

Among women aged 25 to 65 years, CC caused a loss of 2.4 million weighted Years of life lost (YLL) in developing countries, whereas 0.3 million YLL in the developed countries [7]. At late-stage cervical cancer is associated with low survival rates after surgery or radiotherapy, family stress and major income loss [8]. Each year, an estimated 530,000 new cases of cervical cancer are diagnosed globally and more than 270,000 women die from it; around 85% occur in developing countries that lack screening for CC [9]. In developed countries the incidence of cervical cancer has decreased due to effective screening, early detection and treatment [10]; where as in sub-Saharan countries about 80% of CC is incurable at the time of detection due to lack of information and prevention services [8]. Cancer of the cervix accounts for 13.4% of cancers in Ethiopia [11]. In Eritrea, CC is the second most morbidity and mortality causing disease among women between 15 and 44 years of age [12].

According to UN administration for refugee and returnee affairs (ARRA) health information system (HIS) report; as of 2015 the magnitude of VIA positive rate was 11.3%. Cervical cancer is a preventable disease because of a long precancerous stage, effective screening and treatment for precancerous lesions [13]. However, precancerous cervical lesion screening is a new concept for many refugee women and its rates of screening are significantly low [14]. In low-resource settings such as refugee, VIA is sensitive alternative screening method [15] .This is supported by the data from multicentre trials which shows VIA to be moderately sensitive for precancerous cervical lesions compared with Pap testing [16] . VIA is cheap screening method which can be done at lower health facilities by trained staff and its result are available immediately allowing treatment of screened positives the same day [15, 16]. VIA test for screening of cervical cancer has also shown a great deal of promise in cross-sectional studies [17].

Refugee women have a higher risk of cervical cancer because of lower screening rates and high HPV infection [18]. Integrating and mainstreaming of refugees into national and local health services helps to reduce CC death rates through early diagnosis, treatment and HPV vaccination [19].

In Eritrea which is the refugees’ country of origin, cervical cancer screening and early detection by PAP and VIA were not available at public primary health care level [20]. As a strategy In Ethiopia screening with VIA and providing treatment for precancerous cervical lesions with cryotherapy was started initially among HIV positive women; later the service expanded to the general population [8] and in all refugee camps as part of United Nation Higher Commissioner for Refugee (UNHCR) global initiative and in line with the strategic priorities of the Ethiopian Ministry of Health [21].

Even though cervical cancer is recognized as a major public health problem in various sub-Sahara African countries including Ethiopia, there is no clear information on the magnitude and factors associated with precancerous cervical lesions especially among women who are living in the refugee camps. Therefore the aim of this study was to assess the magnitude and factors associated with VIA positive test results for Cervical Cancer Screening among Eritrean refugee women aged 25–49 years in northern Ethiopia refugee camps.

## Methods

### Study area and period

This study was conducted in the Eritrean refugee camps which are located in the northern Ethiopia. There were four Eritrean refugee camps in shire operation. These were Adi harush, shimelba, Mayayni and Hitsats refugee camps which are located 1167 km, 1203 km, 1150 km and 1120 km away from Addis Ababa, the capital city of Ethiopia respectively. According to the UNHCR population data base progress report, there were a total of 39,940 Eritrean refugees in shire operation (24,234 Male, 15,706 Female). Each refugee camp has one health center and one elementary school. The health centers provide outpatient, inpatient, laboratory, pharmacy, mental health, nutrition, maternal and child health services for the refugees. Data were collected from August 10 to September 25, 2018.

### Study design

A community based cross-sectional study design was conducted.

### Study population

All women aged 25–49 years who live in the refugee camps were the source population. The Study population was all sampled refugee women 25–49 years old who had refugee status card. Refugee women who were unable to communicate, had mental health problems, uterine prolapsed, who didn’t start sexual intercourse, suspected of cervical cancer and those with no visible SCJ were excluded from the study.

### Sample size determination

Sample size was calculated using single population proportion formula by taking 50% of population proportion (to maximize sample size since the prevalence of previous studies was very small), 95% CI (Ζ 1-α/2) = 1.96 and 5% margin of error (d).

$$ \mathrm{n}={\mathrm{z}}^2\mathrm{p}\ \left(1-\mathrm{p}\right)/{\mathrm{d}}^2\kern0.75em =(1.96)\ (1.96)\ 0.50\ \left(1-0.50\right)/(0.05) $$

$$ =3.84\ \mathrm{x}\ 0.50\ \mathrm{x}\ 0.50/0.0025\kern0.5em \mathrm{n}=384 $$

Considering 10% contingency the final sample size (n) were = 422.

### Sampling techniques

All refugee camps in northwest Tigray were included in the study. The sample size was proportionally allocated to each refugee camp based on the number of women in the age range of 25–49 years in each camp. In all refugee camps the list of all women in the age range of 25–49 years were registered through home to home visit of community health workers. Study subjects were selected by computer generated random numbers using list of women recorded in the home to home visit as a sampling frame. Selected study subjects were invited to the health institutions for interview and cervical examination.

### Data collection and data quality control methods

Data were collected using structured interviewer administered questionnaire and cervical examination. The questionnaire which consisted of socio-demographic, reproductive and behavioral factors were developed first in English and then translated to the local language Tigrina (Supplementary file 1). The questionnaire was pretested on 5% of the sample size in Endabaguna screening center. After counseling study subjects about the procedure of cervical examination; they were taken to examination couch and then using sterile techniques sterile speculum was inserted to the vaginal canal to visualize the cervix, examine for STI and inspect the SCJ. For study subjects with clear SCJ 3–5% acetic acid were applied into the cervix and waited for 1 min to see the color changes. VIA test result was labeled as VIA positive or VIA negative. VIA positive was defined as presence of raised and thickened white plaques of aceto-white usually near the SCJ after applying 3–5% acetic acid; whereas VIA negative was defined as presence of smooth, pink, uniform and featureless including ectropion, polyp, cervicitis, inflammation, Nabothian cyst. Cryotherapy was applied for those with VIA test positive and who are eligible. Women with suspected cervical cancer were excluded from the study and referred to higher hospitals. For each refugee camp one nurse and 1 medical doctor were recruited for interview and cervical examination respectively. The data collectors were trained for 3 days on how to interview, perform cervical examination using VIA test and provide treatment by cryotherapy. Data collectors who perform cervical examination were blinded to the finding of the questionnaire. For the purpose of this study the outcome variable precancerous cervical lesions was determined by VIA status; VIA test positive corresponded to precancerous cervical lesions. From here in we refer to VIA positive precancerous cervical lesions when describing our results. In this study sexually transmitted infections are defined as having history of purulent vaginal discharge, lower abdominal pain, genital ulceration and risk factors for STI.

### Data processing and analysis

Data entry and cleaning was done using Epi info version 7 and exported to SPSS Version 21 for analysis. Descriptive statistical analyses such as frequencies, percentages, proportion with 95% CI have been used. Median, mean and standard deviation was also used to summarize various characteristics of the respondents. To identify factors associated with VIA positive precancerous cervical lesions; first a bivariable logistic regression was performed. Subsequently, significant variables in the bivariable analysis (*p*-value < 0.25) were incorporated into the multivariable logistic regression.

## Results

### Socio- demographic characteristics of respondents

In this study a total of 412 respondents aged 25–49 years were included making a response rate of 98%. The mean (±SD) age of the respondents was 32.8 (± 5.8) years and 116(28.2%) of them were in the age range of 35–39 years. With regard to the respondents’ marital status; 340(82.5%) of them were married followed by those who were divorced which accounts for 13.3%. Majority of the study subjects 227(55.1%) attended elementary education [Table 1].

**Table 1:** Socio-demographic characteristics of women aged 25–49 years in Eritrean refugee camps, Tigray, northern Ethiopia

Variable	Frequency (n)	Percentage (%)
Age in years (*N* = 412)		
25–29	81	19.6
30–34	93	22.6
35–39	116	28.2
40–44	70	17.0
45–49	52	12.6
Marital status (*N* = 412)		
Single	15	3.6
Married	340	82.5
Divorced	55	13.3
Other^a^	2	0.5
Religion (*N* = 412)		
Orthodox	299	72.6
Muslim	37	9.0
Protestant	17	4.1
Catholic	59	14.3
Educational status (*N* = 412)		
No formal education	110	26.7
Primary education	227	55.1
Secondary education	68	16.5
Collage and above	7	1.7

Other^a^ - Widowed, separated

### Reproductive and behavioral related factors

The mean (±SD) age at first sexual intercourse was 16.9 (± 3.0) years and 316(76%) of the respondents started their first sexual intercourse below the age of 18 years. From the total respondents 274(66.5%) of them were not using contraception methods. Three hundred ninety six (96.1%) of the women had 1–3 (with mean 1.4 (SD ± 0.9)) sexual partner in their life time. All respondents didn’t have family history of cervical cancer and previous screening for precancerous cervical lesions. Four hundred ten (99.5%) and 347(84.2%) of the respondents didn’t have history of cigarette smoking and alcohol drinking respectively [Table 2].

**Table 2:** Reproductive and behavioral factors among women aged 25–49 years in Eritrean refugee Camp, Tigray, Northern Ethiopia

Variable	Frequency (n)	Percentage (%)
Age at first sex (in years)		
< 18	313	76.0
> 19	99	24.0
Previous history of STI (*N* = 412)		
Yes	86	20.9
No	326	79.1
Partner’s STI status (*N* = 412)		
Yes	41	10.0
No	371	90.0
Parity (*N* = 412)		
0–3	273	66.3
4–7	134	32.5
8–10	5	1.2
Sex with uncircumcised male (*N* = 412)		
Yes	24	5.8
No	388	94.2
Number of sexual partner (*N* = 412)		
1–3	396	96.1
4–7	16	3.9
Family history of cervical cancer (*N =* 412)		
No	412	100.0
Yes	0	0
Previous history of cervical screening (*N* = 412)		
Yes	0	0
No	412	100.0
History of cigarette smoking (*N* = 412)		
Yes	2	0.5
No	410	99.5
History of Alcohol drinking (*N* = 412)		
Yes	65	15.8
No	347	84.2
Condom use (*N* = 412)		
Yes	12	2.9
No	400	97.1

### Immune suppressive related factors

From the total respondents, 410(99.5%) of them didn’t have history of Diabetic Mellitus. With respect to the HIV status of the respondents; 270 (65.5%) of them reported as they are HIV negative followed by those who don’t know their HIV status and those who report as HIV positive which accounts for 31.8 and 2.7% respectively.

### Magnitude of VIA positive precancerous cervical lesions

The magnitude of VIA positive precancerous cervical lesions among Eritrean refugee women aged 25–49 years was 9% (95% CI: 6.3–11.8%).

### Factors associated with precancerous cervical lesions

In the binary analysis, variables associated with the outcome variable (variables with *P*-Value of < 0.25) were age of the respondent, self reported HIV status of the respondent, educational status of the respondent, number of sexual partner, previous history of STI, and presence of sign & symptoms of STI during cervical examination. After the multivariable analysis previous history of STI, presence of sign &symptoms of STI during cervical examination were significantly associated with VIA positive precancerous cervical lesions. Women who had previous history of STI were 2.84 times more likely to have VIA positive precancerous cervical lesions as compared to those who didn’t have previous history of STI [AOR (95%CI) = 2.84(1.07–7.53)]. Similarly, presence of sign &symptoms of STI during cervical examination was also found associated with VIA positive precancerous cervical lesions. Women who had sign and symptoms of STI during cervical examination were 3.97 times more likely to have VIA positive precancerous cervical lesions as compared to those who had not [AOR (95%CI) =3.97(1.75–9.00)] [Table 3].

**Table 3:** Bivariable and multivariable analysis of factors associated with VIA positive precancerous cervical lesions among Eritrean refugee women, Tigray, northern Ethiopia

Variable	VIA positive Precancerous cervical lesions		COR (95%CI)	AOR (95%CI)
	Yes N (%)	No N (%)		
Age in years				
25–29	4 (4.9)	77 (95.1)	1	1
30–34	11 (11.8)	82 (88.2)	2.58 (0.789–8.45)	2.14 (0.61–7.53)
35–39	13 (11.2)	103 (88.8)	2.43 (0.762–7.74)	2.57 (0.72–9.19)
40–44	4 (5.7)	66 (94.3)	1.16 (0.28–4.8)	1.39 (0.29–6.64)
45–49	5 (9.6)	47 (90.4)	2.04 (0.52–8.01)	1.65 (0.36–7.63)
Previous history of STI				
Yes	15 (40.5)	71 (18.9)	2.9 (1.44–5.91)	2.84 (1.07–7.53)*
No	22 (59.5)	304 (81.1)	1	1
STI during cervical examination				
Yes	16 (43.2)	50 (13.3)	4.9 (2.4–10.13)	3.97 (1.75–9.00)*
No	21 (56.8)	325 (86.7)	1	
Uncircumcised male partner				
Yes	4 (10.8)	20 (5.3)	2.15 (0.69–6.67)	1.75 (0.49–6.22)
No	33 (89.2)	355 (94.7)	1	1
Self reported HIV status				
Negative	18 (48.6)	251 (66.9)	1	1
Unknown	17 (45.9)	114 (30.4)	2.07 (1.03–4.18)	2.19 (1.01–4.74)
Positive	2 (5.4)	10 (2.7)	2.78 (0.56–13.7)	3.65 (0.62–21.52)
Parity				
0–3	20 (54.1)	253 (67.5)	1	1
4–7	16 (43.2)	118 (31.5)	1.71 (0.85–3.40)	1.79 (0.81–3.95)
8–10	1 (2.7)	4 (1.1)	3.16 (0.33–29.65)	5.73 (0.44–73.08)

## Discussion

The overall prevalence of VIA positive precancerous cervical lesions among Eritrean refugee women in the age group of 25–49 years was 9%. The magnitude of this study is similar with the result of studies done in Nepal (7.1%) [22], USA (9%)&Burmese (10%) [23]. The similarity between these studies might be due to similarity of the study groups which are refugee women; which might have similar behavioral and sexual risk factors. Additionally this similarity might be due to similarity of study subjects’ age limit.

Finding of our study is also similar with the finding of studies conducted in the general population in north Ethiopia (6.7%) [24], WHO study in African countries (Tanzania (9.7%), Uganda (7.8%), Madagascar (11.3%) [25], but higher than the finding of studies conducted among women in Rwanda (5.9%) [26], and Jakarta (4.7%) [27]. Conversely, the current finding is lower than the findings in South west Ethiopia (12.9%) [28], Tanzania (26.8%) [29], Malawi (12.4%), and Zambia (28%) [25]. This discrepancy might be due to difference of study subjects by which the above studies specially the study done in Tanzania is among HIV infected women who mostly had many life time sexual partner and high rate of smoking which accounts 33.6%.

This study revealed that having previous history of sexual transmitted disease was significantly associated with VIA positive precancerous cervical lesions. Women who had previous history of sexual transmitted disease were 2.84 times more likely to have precancerous cervical lesions as compared to those who didn’t have previous history of sexual transmitted disease [AOR (95%CI) = 2.84(1.07–7.53)]. Similarly, women who had signs and symptoms of STI during cervical examination were 3.97 times more likely to have VIA positive precancerous cervical lesions as compared to those who had not [AOR (95%CI) =3.97(1.75–9.00)]. This finding was consistent with the finding of studies done among women in southwest Ethiopia, north Ethiopia, Tanzania and Uganda which showed positive association between sexual transmitted disease and precancerous cervical lesion [24, 28–30]. This can be explained by the role of unprotected sexual intercourse for acquisition of precancerous cervical lesions.

### Limitation of the study

Limitation of this study is lack of histological confirmation of the precancerous cervical lesions; despite its limitations VIA is an accepted cervical screening method in low-resource countries such as Ethiopia. Response to some questions also might have recall and social desirability bias. In addition to this since the number of women who had precancerous cervical lesions was low this might limit the ability to examine association with various risk factors.

## Conclusion

In the study area VIA positive precancerous cervical lesion was high (9%). Previous history of sexual transmitted disease and presence of sign & symptom of STI during cervical examination were significantly associated with precancerous cervical lesions. Efforts such as early screening for sexually transmitted disease shall be done to prevent precancerous cervical lesions.

## Supplementary information

**Additional file 1.** QUESTIONNAIRE.

## Data Availability

The datasets used and/or analyzed during the current study available from the corresponding author on reasonable request.

## Abbreviations

AIDS: Acquired immunodeficiency syndrome
ARRA: Administration for Refugee and Returnee Affairs
CC: Cervical cancer
CIN: Cervical Intraepithelial Neoplasia
DM: Diabetes Mellitus
HIS: Health Information System
HIV: Human Immune Deficiency Virus
HPV: Human Papilloma Virus
MOH: Ministry of Health
STI: Sexual Transmitted Infection
SVA: Single Visit Approach
UNHCR: United Nation Higher Commissioner for Refugee
VIA: Visual Inspection with Acetic Acid
WHO: World Health Organization
