# Supplementary material for: Magnitude and associated factors of VIA positive test results for cervical cancer screening among refugee women aged 25–49 years in North Ethiopia
Source: BMC Cancer. 2020 Sep 7;20:858. doi: 10.1186/s12885-020-07344-9 (PMC7487853; doi:10.1186/s12885-020-07344-9)
Supplement: Supplementary file 1 — Additional file 1. QUESTIONNAIRE. [file 12885_2020_7344_MOESM1_ESM.docx]

**QUESTIONNAIRE:**

**PART 1: SOCIO-DEMOGRAPHIC**

| Code/no. | Questions | Response | Remark |
| --- | --- | --- | --- |
| 101 | What is your age in complete years? | ________ yrs |  |
| 102 | Where is your camp | 1.maiayni 2.adiharush  3.shimelba 4.hitsats |  |
| 103 | What is your Marital status? | 1. Single 2. Married/cohabitate  3. Divorced 4. Others, |  |
| 104 | What is your Educational status | 1.No formal education 2.Primary education (1-8) 3 Secondary education (9-12) 4.college and above (12+) |  |
| 105 | What is your religion | 1. Orthodox  2. Muslim  3. Protestant4. Catholic  5. Others specify |  |
| 106 | What is your Ethnicity | 1. Tigrigna 2. Saho 3. Kunama 4. Tigre 5.blen 6. If other specify |  |

**Part two: reproductive health related variables**

|  |  |  |  |
| --- | --- | --- | --- |
| Code/no. | Question | Response | Remark |
| 201 | Have you ever had History of sexually transmitted infection | 1.Yes 2.No | If your ans is yes respond to202-206 |
| 202 | Do you have history of offensive vaginal discharge | *1.*Yes 2.No |  |
| 203 | Do you have history of Vaginal Itching or Dysuria | *1.*Yes 2.No |  |
| 204 | Do you have history of Lower abdominal pain | *1.*Yes 2.No |  |
| 205 | Do you have history of genital ulceration | 1.Yes 2.No |  |
| 206 | Have you treated for STI symptoms | 1.Yes 2.No |  |
| 207 | Did your Partner have STI history | 1.Yes 2.No |  |
| 208 | Have you ever used contraceptive | *1.*Yes 2.No | If No, go to no.211 |
| 209 | If yes what types of contraceptives did you use? | 1.ocp 2.implanol 3.depo 4.tradatinal methods |  |
| 210 | For how long did take it the contraceptive | ________ yr |  |
| 211 | How many children did you give birth both alive and dead | ________ |  |
| 212 | Do you have known Family history of cervical cancer | *1.*Yes 2.No |  |
| 213 | If yes to question 216, can you tell the degree of relative? | *1.*Mother 2.Sister3.Others |  |
| 214 | Previous history of abnormal cervical screening for precancerous cervical lesion | *1.Yes 2.No* |  |
| 215 | What was your Age at first intercourse | ________ |  |
| 216 | Number of life time sexual partner(s) of yours­­­­­­­­­ | ________ |  |
| 217 | Number of life time sexual partner(s) of spouse or friend | _______ |  |
| 218 | Do you have any history of sexual intercourse with uncircumcised men | 1.yes 2.no |  |

**Part three: behavior related variables**

| Code /no | Question | Response | Remark |
| --- | --- | --- | --- |
| 301 | Did use Condom during sexual intercourse persistently and correctly | *1.*Yes 2.No |  |
| 302 | Do you have self-history of Smoking: | *1.*Yes 2.No |  |
| 303 | If yes to question 303, duration? | _______years |  |
| 304 | If yes to question 303, amount? | _______packs |  |
| 305 | If your answer to Q303 is no, is there any smoker living in your house | 1.yes 2.no |  |
| 306 | If yes to question 306,for how long lived together | _______years |  |
| 307 | Did you take Alcohol | 1. Yes 2.No |  |
| 308 | If yes to Q308, what type? | 1. Local alcoholic drink 2.beer 3.waine 4.heavy alcohols |  |

**Part four: immunity related variables**

| Code/no | Question | Response | Remark |
| --- | --- | --- | --- |
| 401 | Do you have history of type 1or type 2 DM | *1.*Yes 2.No |  |
| 402 | Do you have history of Asthma with corticosteroid therapy | *1.*Yes 2.No |  |
| 403 | Do you have Chronic corticosteroids use | *1.*Yes 2.No |  |
| 404 | Do you have History of chemotherapy for any cancer | 1. *Yes 2. No* |  |
| 405 | Do you have History of radiotherapy for any cancer | 1. *Yes 2. No* |  |
| 406 | What is your HIV status | 1.Positive 2.Negative 3.Unknown |  |
| 407 | If your ANS to question 409 is positive, your current CD4 count | CD4 count________ |  |
| 408 | If your ANS to question 409 is positive, are u on ART? | *1.yes 2.no* |  |
| 409 | weight | _______kg |  |
| 410 | height | _______cm |  |
| 411 | BMI | _______ |  |

**Part five: clinical examination**

| Code /no | Question | response | remark |
| --- | --- | --- | --- |
| 501 | Is there active lesion for STI during examination | 1.yes 2.no |  |
| 502 | Is SCJ completely seen | 1.Yes 2.No |  |
| 503 | What is VIA Result: | 1. Suspicious for Cancer 2.Negative 3. Positive |  |
| 504 | If VIA positive, Cryotherapy details | 1. Done immediately (same day) 2.Done other day 3.Refused Cryotherapy  4.Ineligible for Cryotherapy |  |
| 505 | If client was referred, Reasons of referral | 1. Suspicious for cancer  2.Lesion larger than cryoprobe>2 mm  3.Client denied cry therapy 4.Lesion > 75%  5. Lesion extended inside cervical os  6. PID 7. Other non-gynecologic or gynecologic problem |  |

Providers: Name__________________________ Signature______________________
